# Influences of sex and gender on the associations between risk and protective factors, brain, and behavior

**DOI:** 10.1186/s13293-024-00674-4

**Published:** 2024-11-26

**Authors:** Katharina Brosch, Elvisha Dhamala

**Affiliations:** 1 Institute of Behavioral Science, Feinstein Institutes for Medical Research, Manhasset, NY USA; 2 Division of Psychiatry Research, Zucker Hillside Hospital, Glen Oaks, NY USA; 3 Donald and Barbara Zucker School of Medicine at Hofstra/Northwell, Uniondale, NY USA

**Keywords:** Sex differences, Gender differences, Risk factors, Resilience, Protective factors, Major depressive disorder, Brain imaging

## Abstract

Risk and protective factors for psychiatric illnesses are linked to distinct structural and functional changes in the brain. Further, the prevalence of these factors varies across sexes and genders, yet the distinct and joint effects of sex and gender in this context have not been extensively characterized. This suggests that risk and protective factors may map onto the brain and uniquely influence individuals across sexes and genders. Here, we review how specific risk (childhood maltreatment, the COVID-19 pandemic, experiences of racism), and protective factors (social support and psychological resilience) distinctly influence the brain across sexes and genders. We also discuss the role of sex and gender in the compounding effects of risk factors and in the interdependent influences of risk and protective factors. As such, we call on researchers to consider sex and gender when researching risk and protective factors for psychiatric illnesses, and we provide concrete recommendations on how to account for them in future research. Considering protective factors alongside risk factors in research and acknowledging sex and gender differences will enable us to establish sex- and gender-specific brain-behavior relationships. This will subsequently inform the development of targeted prevention and intervention strategies for psychiatric illnesses, which have been lacking. To achieve sex and gender equality in mental health, acknowledging and researching potential differences will lead to a better understanding of men and women, males and females, and the factors that make them more vulnerable or resilient to psychopathology.

## Abstract (significance)

Exposure to different environments and distinct lived experiences for individuals across the sexes and genders have unique effects on mental wellbeing, brain structure, and function. These differences in outcomes arise from interdependent effects of biology and socio-culture and can be challenging to separate. This review describes sex- and gender-specific effects of negative and positive environmental experiences, known as risk and protective factors. Specifically, we review current sex- and gender-specific findings for childhood maltreatment, the COVID-19 pandemic, experiences of racism, social support, and resilience. Risk and protective factors, and sex and gender have bidirectional influences, and our review outlines their dynamic, interconnected and intersectional nature. Including sex and gender as a variable of interest will enable us to capture specific effects that may have previously gone undetected. More broadly, implementing the outlined considerations will lead to more inclusive and representative research that can deliver robust findings and meaningfully propel the field forward.

## Highlights

Unique effects of sex and gender on brain and behavior necessitate the investigation of these factors as a variable of interest in research.

Protective factors should be considered alongside risk factors for a more holistic understanding of mental wellbeing.

Investigating sex and gender influences will help detect findings that would have otherwise been overlooked.

Risk factors, protective factors, sex, and gender all have bidirectional influences on each other.

## Background

Risk and protective factors for psychiatric illness are associated with structural and functional changes in the brain. As an example, childhood maltreatment is one of the most prominent risk factors. It is associated with an up to 3.37 times increased risk of developing major depression, along with changes in gray matter volume, cortical thickness, and functional brain connectivity [1–3]. Other critical environmental risk factors include stressful life events, such as the COVID-19 pandemic, and experiences of racism. On the other hand, protective factors, such as social support and psychological resilience, decrease an individual’s likelihood of developing psychiatric disorders and exert independent effects on the brain [4–6]. Specifically, protective factors have been linked to alterations in brain regions associated with emotion regulation, cognitive flexibility, and behavioral control [7, 8]. However, risk and protective factors do not exist in isolation. Rather, they interactively influence both brain and behavior and as such, should be jointly considered in research and clinical practice. To further complicate matters, different risk and protective factors vary between men and women, and males and females. For example, while one in 5 females report experiences of childhood sexual abuse, a form of childhood maltreatment, it is reported by one in 13 males worldwide [9]. Females also report higher levels of social support and help-seeking behavior, while males reported higher psychological resilience levels [10–12]. Therefore, different risk and protective factors may uniquely or disproportionately impact individuals across sexes and genders [13]. Taken together, this further highlights the need to consider sex and gender when investigating the influences of risk and protective factors on brain and behavior.

Previously, the term “sex” has been used to describe biological influences, while “gender” was used to describe sociocultural influences. However, this distinction does not account for the bidirectional effect of biological and sociocultural influences [14]. These effects may, in turn, influence variability in the brain and impact the emergence and presentation of psychopathology [14]. To emphasize that sex influences gender, and vice versa, and in line with prior literature, we use the term “sex and gender” throughout this manuscript. This terminology aims to highlight the fact that complex human behaviors may be influenced by distinct and interrelated effects of sex and gender, and their effects may be difficult to disentangle [15]. Although gendered terms (i.e., “men and women”) may be used in the original articles that we review here, we use the terms “female” and “male” when referring to research on sex differences to more accurately describe the information that was collected about participants (e.g., self-reported sex). We encourage readers to refer to the original articles for more details. Critically, we note that little research is available specifically on gender effects, and previous literature investigating differences between “men and women” may depict both effects of sex and gender. Additionally, we focus on extant literature evaluating differences in binary sex and gender but note that these terms do not include all sexes or genders (both of which are non-binary in nature). Importantly, we focus on exemplary environmental risk factors, but note that important genetic risk and protective factors exist which are beyond the scope of this review [16–18].

Sex effects encompass the influences of genes, hormones, immune responses, and stress responses. This includes health conditions that only affect individuals who menstruate (e.g., postpartum depression, polycystic ovary syndrome, PCOS) [19], and factors such as the use of hormonal birth control, which may be linked to depression [20]. Conversely, the effects of gender entail influences of gender roles and stereotypes. Specifically, sociocultural expectations impact how individuals express, communicate, and cope with mental health issues [11]. In patriarchal societies, rules exist for what are considered appropriate “male” and “female” expressions and behaviors. Non-conforming to these rules may make integration in a peer group more difficult. As an example, traditional patriarchal gender roles view expressions of vulnerability as feminine and less desirable for men to express. In Latin America, “Machismo Culture” describes a rigid set of behavioral norms for men that encompass rules for favored behaviors and character traits [21]. Patriarchal societies may share overlap in such gender stereotypes but influences on the individual can vary by country and more microlevel settings such as peer group, city, or neighborhood.

Studies have shown that sex and gender exert unique influences on behavior and distinctly influence outcome domains (e.g., cognition) [22]. Regarding brain outcomes, it was demonstrated that sex and gender are associated with distinct functional connectivity networks. This study included *N* = 4,757 children (2,315 female, 2,442 male) aged 9–10 of the Adolescent Brain Cognitive Development (ABCD) cohort [14]. These findings underscore the need to consider both constructs of sex and gender in research. In humans, the effects of sex and gender on behavior and the brain are highly intertwined. As an example, depressive symptoms are more frequently reported by women. This finding has been attributed to biological and sociocultural differences. Biologically, sex-specific gene expression patterns in the brain related to depression have been reported [23]. Socioculturally, norms around the expression of depressive symptoms differ. 

 The sociocultural stigma around individuals with a masculine identity expressing feelings of sadness may lead to them expressing distress in more externalizing ways, such as binge drinking. Additionally, lower help-seeking behavior and a social desirability bias may lead to underreporting and non-detection of depressive symptoms in individuals with a masculine identity [11].

This example illustrates that behavioral outcomes are shaped by multifactorial influences, where biological factors are always embedded in a cultural context, highlighting the complexity of human behavior and the need to move beyond monocausal explanations [15].

Here, we aim to describe how risk and protective factors independently and interdependently impact neurobiology and mental health across sexes and genders. We focus on prominent risk factors such as childhood maltreatment, racism, and the COVID-19 pandemic that have recently been the focus of significant attention in both research and public policy [24–27]. We highlight why it is essential to consider protective factors, such as social support and psychological resilience, in future research to adequately support individuals at risk of developing psychiatric disorders. Finally, we provide concrete recommendations on how to consider risk and protective factors and their sex and gender-specific effects meaningfully in future research.

## Main text

### Sex- and gender-specific effects of risk factors on mental health and the brain

Risk factors for psychiatric illness and their impact on behavior, along with brain function and structure, have been studied extensively. Risk factors span biopsychosocial aspects and include childhood maltreatment, stressful life events, loneliness, neuroticism, and familial and genetic risk, to name a few [18, 28–31]. While certain disorder-specific risk factors have been identified, many risk factors (e.g., childhood maltreatment and stressful life events) are transdiagnostic and broadly increase the risk for all psychopathology [27–29]. Here, we discuss how the influences of risk factors on mental health and the brain differ across sexes and genders.

### Childhood maltreatment

Childhood maltreatment encompasses emotional and physical neglect as well as emotional, physical, and sexual abuse. These forms of childhood maltreatment often co-occur, and several studies have investigated their independent and joint influences on brain and behavior [32–34]. Across sexes and genders, exposure to childhood maltreatment is causally linked to an increased risk of developing psychopathology (e.g., major depression, anxiety, substance abuse) [35–38].

In the brain, a history of childhood maltreatment, even in individuals with no psychiatric illnesses, is associated with structural alterations that are similar to those found in individuals with depression [35, 39]. Childhood maltreatment is also negatively associated with gray matter volume and cortical thickness in the median cingulate and paracingulate gyri [2]. Additionally, smaller gray matter volumes in the hippocampus, prefrontal cortex, anterior cingulate, and left supplementary motor area, as well as cortical thinning in the right anterior cingulate gyri and left middle frontal gyrus, have been reported in individuals with a history of childhood maltreatment [2, 38, 39]. These regions are implicated in emotion and stress regulation. Taken together, this suggests that childhood maltreatment may impair emotion and stress regulation, as well as emotion integration, and predispose individuals to psychiatric illness [2, 38].

Childhood maltreatment has also been associated with altered resting-state functional connectivity. A recent systematic review including *n* = 3079 individuals identified reduced connectivity between the dorsal anterior cingulate cortex and anterior insula and heightened amygdala connectivity in the salience, default mode, and prefrontal regulatory networks in individuals with a history of childhood maltreatment. Altered functional connectivity was further reported in the ventral anterior cingulate cortex, dorsolateral prefrontal cortex, and hippocampus [40]. These regions are associated with emotional reactivity, salience detection, fear conditioning, autobiographical memory, and reinforcement-based learning, suggesting potential impacts on these behaviors. While these alterations in functional connectivity may be regarded as short-term adaptations to highly adverse environments, they may predispose individuals to future psychopathology over time [41].

**Fig. 1:**
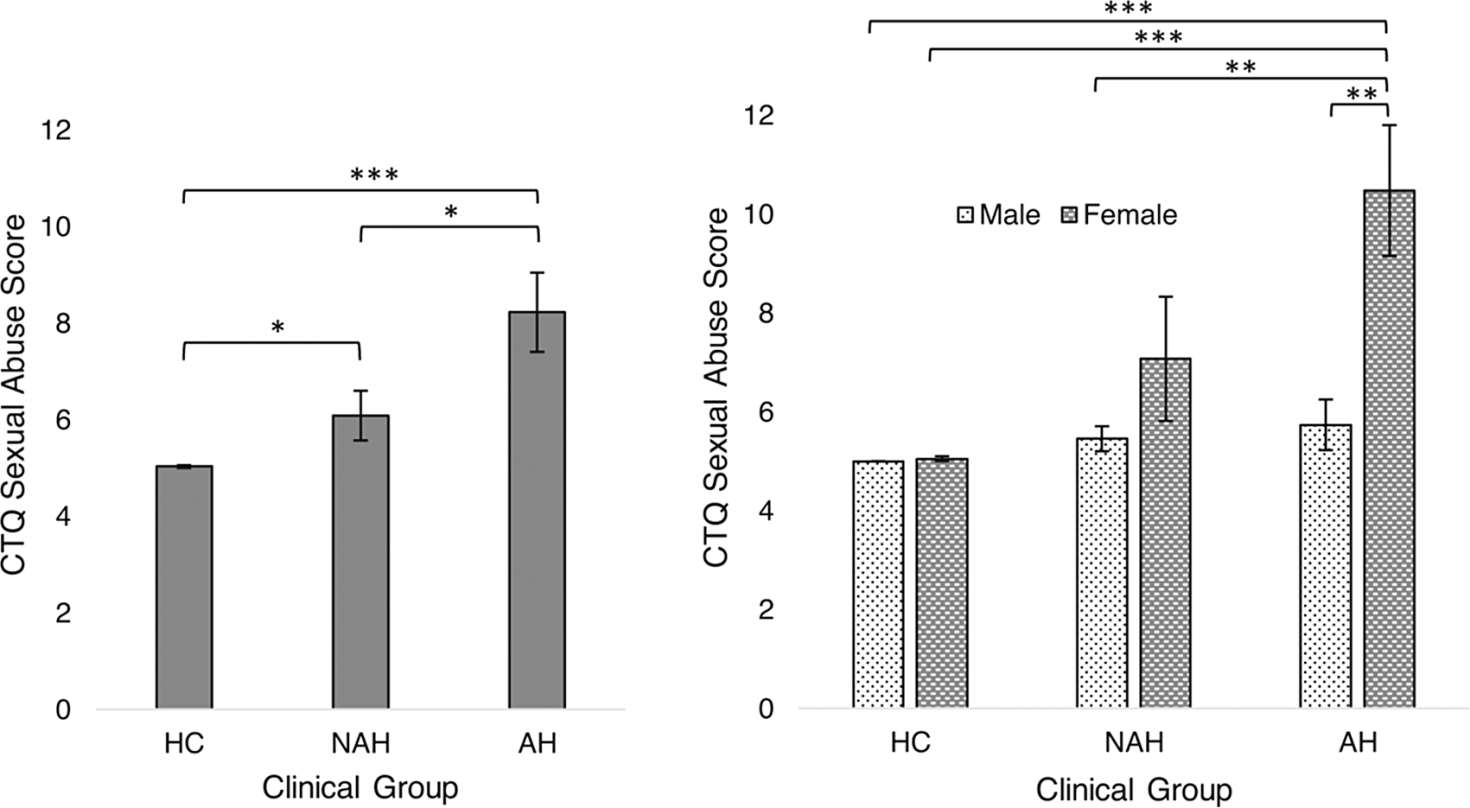
Sex-specific analyses reveal a higher prevalence of childhood sexual abuse in females, but not males, with auditory hallucinations (AH). This study investigated the influences of sex and childhood sexual abuse in individuals with auditory hallucinations. They found that individuals with auditory hallucinations were more likely to report childhood sexual abuse, relative to individuals with non-auditory hallucinations and healthy controls. Further sex-specific analyses found that these effects were driven entirely by females, and were absent in males. These results underscore the critical importance of sex-specific analyses in biomedical research Figure reprinted from “Auditory hallucinations, childhood sexual abuse, and limbic gray matter volume in a transdiagnostic sample of people with psychosis” by Millman et al., 2022 [43]: *Childhood sexual abuse exposure across clinical group and sex. Differences in exposure severity between (A) AH*, *NAH*, *and HC groups and between (B) AH*,*NAH*, *and HC groups by participant sex. Error bars represent standard deviations. AH*, * psychotic disorder with auditory hallucinations; CTQ childhood trauma questionnaire*, *HC healthy control*, *NAH psychotic disorder with no auditory hallucinations.* *p < .05, **p < .01, **p < .001. https://creativecommons.org/licenses/by/4.0/

Critical sex and gender differences have been identified in the prevalence of specific subtypes of childhood maltreatment and their behavioral influences. Exposure to childhood sexual abuse is reported by one in 5 females and one in 13 males [32]. Although there is limited evidence thus far demonstrating sex and gender differences in the effects of childhood maltreatment on the risk of developing psychopathology [30], sex and gender effects have been shown to moderate the effect of childhood trauma on depressive symptoms in adulthood [42]. In a study investigating how distinct forms of abuse were associated with depressive symptoms in 560 young adults (223 male, ages 18–20), different trends were observed in males and females. In females, peer emotional abuse at age 14 was the strongest predictor for depressive symptoms, whereas in males, non-verbal emotional abuse at 14 emerged as the strongest predictor [42]. A transdiagnostic study of adults with psychosis revealed distinct behavioral associations with childhood maltreatment across the sexes and genders [43]. Specifically, individuals with auditory hallucinations reported significantly higher amounts of childhood sexual abuse s (*n* = 41),  compared to individuals without auditory hallucinations (*n* = 37) or healthy controls (*n* = 37). However, when exploring this outcome for males and females separately, the authors demonstrated that this difference was entirely driven by the females in the group, who uniquely reported higher scores of childhood sexual abuse (Fig. 1). These findings highlight the importance of including sex- and gender-specific analyses.

Sex and gender may also influence the effects of childhood maltreatment on the brain [43, 44, 33, 34, 45]. Two studies investigating female survivors of childhood sexual abuse found that sexual abuse was associated with specific differences in brain structure, including smaller gray matter volume in the visual cortex and smaller cortical thickness in the primary somatosensory cortex, representing the clitoris and genitalia [33, 34]. Additionally, a meta-analysis (including 38 original articles and *N* = 1042 individuals) examining global effects of childhood maltreatment on the brain reported significant sex and gender effects in the right amygdala, right dorsolateral prefrontal cortex, and right postcentral gyrus. Effect sizes were significantly larger in studies that included more males [44]. Finally, a separate mega-analysis by the ENIGMA consortium including *N* = 3,872 participants reported a sex and gender effect where childhood maltreatment severity was positively associated with higher cortical thickness of the rostral anterior cingulate cortex in males. In females, this effect was not detected, but a global *negative* association between childhood maltreatment and cortical thickness was observed [45]. These findings suggest that brain regions are differentially affected in males and females who experience childhood maltreatment.

Taken together, these studies offer compelling evidence that sex and gender effects exist in childhood maltreatment and its subsequent effects on the brain and psychopathology. These differences may arise due to sex and gender differences in the types of maltreatment experienced and in the trajectories of brain development [41, 44]. As such, it is crucial to implement sex- and gender-specific analyses in this research, as failing to do so may lead to incorrect conclusions.

### Stressful life events

Stressful life events include changes in an individual’s life, such as marriage, illness or job change. While certain stressful life events constitute normal steps in development (e.g., starting college, moving out), others, such as experiences of bodily harm or loss, are inherently traumatic. Greater incidences of stressful life events are causally linked to major depression, and the risk to develop major depression is increased five-fold one month after stressful life events [46]. Specific examples of stressful life events we discuss below are the COVID-19 pandemic and experiences of racism [47–52].

Stressful life events are associated with changes in brain and behavior. In the brain, in both males and females, experiencing higher numbers of stressful life events is linked to smaller volumes in the left hippocampus, left medial prefrontal cortex, and left medial orbitofrontal cortex in cross-sectional and longitudinal designs [53–55]. Further, a meta-analysis of 83 task-based fMRI studies with 5,242 participants found that adverse life experiences were associated with higher amygdala reactivity and lower prefrontal cortical reactivity [56]. In these studies, cumulative scores were used that included the number of self-reported stressful life events and a subjective impact rating of the events. While specific differences between the sexes were not evaluated in these studies, there are differences in the exposure, frequency, and type of stressful life events experienced by males and females [57]. Moreover, males and females differ in terms of their perceived stress and sensitivity in response to stressful events [57]. A study including Caucasian adult twin pairs found that males reported more work-related and legal stressors while females reported more interpersonal stressors [57]. In the same study, males and females also differed in their sensitivity to these events and the types of events that increased their risk for depression. In males, a higher depressogenic effect was found for separation/divorce and work problems, whereas females reported higher sensitivity to social problems in their proximal networks [57]. This suggests that sex and gender are important factors to consider when investigating the influences of stressful life events. Ignoring them may lead to inaccurate risk predictions and research findings, as different outcomes may be observed across sexes and genders.

### Racism

Racism, or race-based traumatic stress, describes the cumulative psychological injury caused by hate of a person due to their race. These experiences, along with discrimination related to other aspects of a marginalized identity (e.g., ethnicity, skin color), negatively affect mental health and are associated with changes in the brain [58]. This discrimination can take multiple forms including physical and verbal abuse, hurtful social practices, as well as microaggressions. Racial microaggressions are subtle, racially motivated maltreatments, insults, or invalidations. Over time, they can have detrimental effects on mental health [59]. Race-based traumatic stress is similar to chronic social stress, as it entails aspects of social rejection and has been associated with chronically elevated cortisol and a dysregulation of the hypothalamic-pituitary-adrenal axis [60–62]. However, research indicates that the effects of racism extend far beyond the impact of proximal traumatic stress. Further pathways which link racism to adverse health outcomes may include transgenerational and prenatal factors, moral injury, and negative affective states stemming from racist cognitive schemata [63–65]. Even when individuals are unaware of their exposure to systemic and institutional racism, it can affect health outcomes: A study analyzing data from *n* = 18,067 psychiatric patients demonstrated that Black, Hispanic/Latinx, and Asian patients were significantly less likely to be assigned to a newer building within a psychiatric hospital. The newer building had better resources, more natural light, bright open areas, and calming interior design choices [66]. These findings compellingly underscore the necessity of a national solution to this health crisis [66, 67].

Experiences of racism cannot be generalized across races and ethnicities and may critically depend on specific environmental factors. In a study of *N* = 10,354 children aged 10–11 in the ABCD dataset, 4.8% of children reported perceived racism. Importantly, 10% of Black children reported perceived racism [68]. Overall, children from lower-income households (≥ $75,000 median annual household income) were more likely to report race or ethnicity-based discrimination. The opposite was true for Black children: living in higher-income households was associated with 8.23 higher odds of perceives racism, compared to 2.43 higher odds for Black children living in lower-income households [68]. This study illustrates the need for the granular description and investigation of race. Researchers should avoid comparisons between White vs. “Non-White” individuals and describe effects on specific races and ethnicities whenever possible. A seminal guideline for the ethical handling of race and ethnicity in neuroscience research can be found here [69].

The “Adultification Bias” describes the alarming finding that Black female children are perceived as more adult-like compared to White female children, with differences emerging as early as age five and peaking at ages 10–14. In a study with over 300 participants, Black female children were seen as less in need of support, nurturing, comfort, and protection [70]. If children are wrongly perceived as more mature, they are treated less leniently and punished more harshly [71, 72]. Indeed, Black female children are disproportionately more disciplined in school [70]. In a study in *N* = 342 adults investigating justification of police use of force, Black children were dehumanized more than White children. Further, Black female children were perceived as less victimized compared to White female children, but also Black male children. The racial identity of the study sample was 82.2% White, 7.8% Asian, 2.9% Black, 0.3% Caribbean, 2.1% Hispanic (2.05%), and 3.5% multiracial [71]. While this study found an intersectional effect of sexism and racism, other studies have suggested that Adultification is also present in Black male children [73]. Adultification bias is dehumanizing and, in effect, robs Black children of their childhood [70], which is expected to influence both neurobiological and behavioral development.

There are also differences in brain function and structure in response to threat between Black and White adults in the US.. Specifically, lower functional activation in the hippocampus, amygdala and prefrontal cortex has been observed in Black Americans (*n* = 143) relative to White Americans (*n* = 55) in response to threatening stimuli. This blunted reactivity may be a predisposing factor for post-traumatic stress disorder or other psychopathology [61]. In terms of brain structure, Black children (*n* = 1,786) exhibited greater gray matter volume in the pars triangularis and smaller gray matter volume in the amygdala, hippocampus, frontal pole, superior frontal gyrus, lateral orbitofrontal cortex, caudal middle frontal gyrus, caudal anterior cingulate gyrus compared to White children (*n* = 7,350) in a study in the ABCD study [60, 61]. These regions have been associated with trauma- and stress-related disorders and alterations may reflect the toxic stress Black children are exposed to. Unfortunately, both studies did not investigate sex and gender effects. However, in both studies, when accounting for stressful life events and childhood adversity, differences between the groups decreased, suggesting these differences may be driven by stress and adversity rather than race itself [55, 56]. Race as a social construct may therefore serve as a proxy for disproportionately higher stress exposures experienced by persons of color due to systemic racism. These stress exposures, in turn, could lead to hormonal and neurobiological changes that increase hypervigilance and contribute to adverse mental health outcomes [74].

Acknowledging the intersectionality of risk factors is especially important in this field of research. Multiple studies have shown how effects of race, and sex and gender can lead to more adverse outcomes [75]. In a recent study investigating white matter integrity in 79 Black American females, fractional anisotropy in the corpus callosum genu mediated the association between racial discrimination and physical health, even after accounting for trauma exposure and socioeconomic status [76]. Another study investigated racial discrimination and cortical thickness in 81 Black trauma-exposed females. Here, higher experiences of racial discrimination were associated with lower cortical thickness in the posterior cingulate cortex and the left rostral and caudal anterior cingulate cortex [77]. These findings highlight the interconnected nature of the influences of experiences of racism and gender on brain and behavior. Critically, both studies did not include men. However, the lack of research in this specific population is not sufficient to conclude that there is no effect. Rather, it highlights the importance of including diverse populations in research and considering the interconnected influences of sex, gender, and race.

### COVID-19 pandemic

The COVID-19 pandemic constitutes a global stressor that spans adverse effects of viral infection and psychosocial stressors.

Post-COVID condition, also referred to as Long-COVID, is defined as persistent or new symptoms after three months after the initial SARS-CoV-2 infection, with symptoms lasting at least 2 months. This condition is estimated to affect 10% of individuals infected [78, 79]. While the etiology of this debilitating condition is unclear, females are more likely to be affected than males [78, 80]. In a neuroimaging study including more than 67% females with post-COVID (total sample *N* = 86), the condition was associated with reduced connectivity between the parahippocampal gyri, and between bilateral orbitofrontal and cerebellar regions [81]. However, no sex-specific analyses were conducted in this study, and it remains to be established if these findings may differ for males and females. Risk for post-COVID condition is higher for females, individuals with lower socioeconomic status, and for individuals unable to rest during acute COVID infection. Concurrent environmental stressors of lockdowns during the pandemic contribute to the overall risk of developing post-COVID condition [78].

Apart from the direct virus-related impacts, sociocultural changes associated with lockdowns and changes in daily routines, health-related concerns, and governmental regulations affected the global population. The individual impact of these changes differed drastically for men and women, and males and females. The pandemic is considered to have exacerbated the gender gap, with females more likely to report loss of employment, forgoing work to care for others, and more female children dropping out of school [82]. Multiple studies also identified females to be at higher risk for worse mental health outcomes during the pandemic [83–86]. As such, it is anticipated that longer-term outcomes of the COVID pandemic, both in terms of brain and behavior, may uniquely influence individuals across sexes and genders.

Further studies are needed to describe long-term effects for mental health and associated outcomes (education, physical health, poverty) of this global viral and social stressor across sexes and genders.

### Sex- and gender-specific effects of protective factors on mental health and the brain

Protective factors mitigate the risk to develop psychopathology and increase positive affect and life satisfaction. They encompass multiple levels, such as individual, family, school, community, and organizational levels [87], and include secure attachment styles, regular physical activity, meditation, religion, specific personality traits (e.g., openness, extraversion, and conscientiousness), along with social support and psychological resilience [88, 89]. In line with the WHO definition of health, mental health is not just the absence of pathology, but rather a state of thriving and “complete physical, mental and social well-being”. Research focusing on the positive effects of protective factors has therefore also focused on outcomes such as life satisfaction and positive affect [90, 91]. Significantly fewer research studies have been done on protective factors, relative to risk factors, and consequently, less is known about sex and gender specific effects on protective factors. Here, we will focus on the effects of social support and psychological resilience on brain and behavior as these have been more widely researched and highlight how potential sex and gender effects may emerge.

### Social support

Social support describes a social network encompassing family, friends, peers, neighbors, and community that may offer different forms of help and support [92]. Social support is associated with positive physical and psychological mental health outcomes and greater life satisfaction [4, 90]. An analysis summarizing findings from 23 meta-analyses including a collective 1,458 million participants demonstrated how social support is associated with longevity. Effect sizes for health outcomes ranged from 0.15 to 0.41, and -0.20 to -0.63 for disease variables [93]. The protective effect of social support presumably works through a stress-buffering effect of human interaction and connection [93]. Importantly, social isolation or loneliness, at the opposite end of the spectrum of social support, constitute a strong risk factor for premature morbidity [29].

Males and females differ in their reliance on social networks and friends. In a study investigating middle-aged to elderly individuals, females (*n* = 166) reported larger social networks and received social support from several people, whereas males (*n* = 214) generally relied solely on their spouses [10]. In this study, only biological sex was assessed, but the found differences may be due to effects of gender, or sex and gender. Specifically, sociocultural expectations around help-seeking behaviors may impede individuals with a masculine identity from reaching out to others and getting adequate social support. Social support is beneficial for all sexes and genders and is associated with significantly decreased mortality in individuals [29]; however, in females, social support has a larger contribution to self-reported happiness than it does for males [10]. In the brain, greater self-reported social support is linked to higher white matter integrity in the corpus callosum [94] and greater gray matter volume in the posterior cingulate cortex, bilateral lingual cortex, left occipital lobe and cuneus in both males and females [95]. Therefore, even though the influences of social support on the brain may be shared across sexes and gender, the distinct behavioral influences seen in the sexes and genders suggests social support may uniquely impart a protective effect against psychiatric illnesses.

### Psychological resilience

Psychological resilience, or “trait resilience”, describes the self-reported ability to maintain or quickly recover mental health despite facing adversity. It encompasses interpersonal factors such as optimism, sense of coherence, coping skills and the ability to find individual meaning [87], and is associated with more positive affect and higher life satisfaction [91, 96].

Items to measure this construct include “I usually come through difficult times with little trouble”, or “My life has meaning” [97, 98]. Psychological resilience can be assessed using self-report questionnaires such as the Connor-Davidson Resilience Scale (CD-RISC), Brief Resilience Scale (BRS), or the Resilience Questionnaire (RS-25) [97–99].

Sex and gender differences in resilience have been reported in a sample of 231 adolescents (121 female), where self-reported psychological resilience was higher in males. Moreover, the association between psychological resilience and gray matter volume was reversed across sexes and genders: in females, higher resilience was associated with smaller volume in the left ventrolateral prefrontal cortex, whereas a positive association was present in males [12]. The authors postulated that differences may be driven by sex differences in the stress system, specifically the hypothalamic-pituitary-adrenal axis (HPA), and in the trajectories of brain maturation during adolescence [12]. Specifically, the authors argued that sex differences in HPA-axis responsivity may be modulated via differences in gonadal steroids and cortisol. Further, brain maturation in males in the prefrontal cortex is linear, whereas an inverted U-shape is reported in females. However, concurrent gender (or sex and gender) effects may also be at play, as males tend to overestimate their own abilities [100].

Regarding brain structural correlates, psychological resilience is positively associated with cortical thickness in the lateral occipital cortex, the fusiform gyrus, the inferior parietal cortex, and the middle and inferior temporal cortex [101].

Importantly, the term “resilience” is also used to indicate the maintenance of mental health despite adversity. In this context, resilience as an outcome describes a favorable mental health outcome from the interplay of certain risk and protective factors. For example, individuals are described as “resilient” if they do not develop PTSD after being exposed to a traumatic event [102]. We describe sex and gender differences and brain correlates of resilience as an outcome below in the section “interdependent effects of risk and protective factors”.

### Interdependent effects of risk and protective factors

Risk and protective factors do not exist in isolation but rather co-occur and interact in a complex manner to influence mental health and neurobiology. It is therefore essential to assess and investigate both factors to examine their effects on brain and behavior.

### Interactive effects

**Fig. 2:**
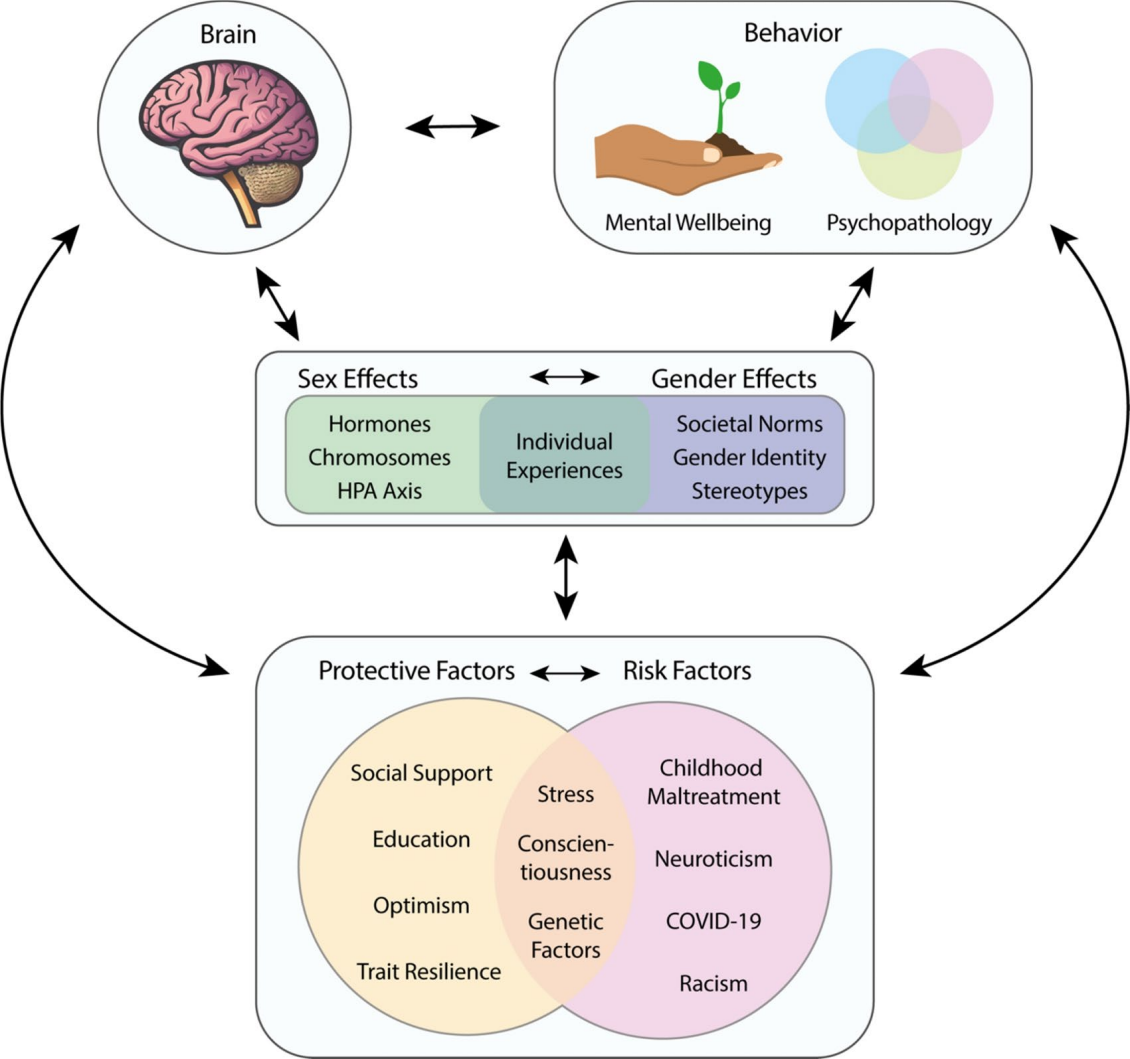
Sex and gender influence associations between brain, behavior, and risk and protective factors. This simplified illustration highlights the complex relationships that exist between brain, behavior, and risk and protective factors and demonstrates how sex and gender may influence those relationships. Sex effects can include influences of hormones, chromosomes, and the HPA (hypothalamic-pituitary-adrenal) axis while gender effects can include the influences of societal norms, gender identity, and stereotypes. Moreover, individual experiences in day-to-day life are influenced by both sex and gender effects. Collectively, these sex and gender effects can influence brain, behavior (in terms of both mental wellbeing and psychopathology), and risk/protective factors, as well as the relationships between then. Moreover, risk/protective factors can be context-dependent and dynamic

Protective factors can exert “rescue effects” and counteract or mitigate adverse effects. As an example, social support has been shown to mitigate the adverse effects of childhood maltreatment and the COVID-19 pandemic [83, 94, 103–105]. During the COVID-19 pandemic, in a study of more than 69,000 participants, social support was associated with 55% lower odds of depression [105]. These influences of risk and protective factors are not exclusively subtractive/antagonistic in their effect on psychopathology (i.e., risk factors increasing the risk, and protective factors decreasing it). Instead, these influences are are likely more complex, with distinct factors acting as moderator or mediator variables [5, 31, 87]. A moderator variable weakens or strengthens the association between two other variables. When a third variable explains the association between two other variables, it is referred to as mediation [106]. Figure 2 demonstrates how sex and gender may influence the interconnected associations between brain, behavior, and risk and protective factors.

A recent study reported a moderation effect in the relationship between childhood maltreatment and social support and their joint impact on gray matter volume. In 181 adults with childhood maltreatment, social support was negatively associated with hippocampal volume, whereas a positive association was detected in individuals without childhood maltreatment (*n* = 265) [107]. Moreover, optimism and distress tolerance have been shown to moderate (i.e., mitigate) the adverse effects of ethnic discrimination in 200 Hispanic individuals [108]. In 223 Black females, self-care mediated the relationship between higher stress and worse health [109]. These findings are examples of the types of non-linear associations that exist between risk and protective factors, and their potential effects on brain and behavior.

While protective factors can offset or mitigate risk factors, risk factors can also compound and jointly exacerbate mental health outcomes. As an example, in minoritized ethnic or racial groups, greater adverse childhood experiences are reported. This is especially true when comparing Indigenous/Native Americans to White Americans [26]. The idea that “adversity breeds adversity” has been termed “double disadvantage” to describe cumulative effects of two or more risk factors. The compounding of multiple forms of marginalization or risk factors exceeds mere additive effects, leading to even worse mental and physical health outcomes [110, 111].

The compounding effect of marginalized experiences and risk factors has also been demonstrated in seminal work investigating the effects of the COVID-19 pandemic. Specifically, a data-driven study including more than 9,200 adolescents and over 17,000 variables found that minoritized populations experienced higher burden during the pandemic [112].

### Resilience as an outcome

Resilience as an outcome can also be operationalized as the absence of psychopathology in the presence of high risk or adversity [113]. Definitions and operationalizations of resilience vary, but most researchers agree that it constitutes an adaptive process in response to adversity [114].

Specifically, one operationalization could be the absence of post-traumatic stress disorder or major depression after trauma exposure (e.g., childhood maltreatment, natural catastrophe, experiences of war, terrorist attacks). In healthy, at-risk individuals (with childhood maltreatment and familial risk), studies identified larger gray matter volume in the left dorso-lateral prefrontal cortex, higher fractional anisotropy in the forceps minor, and right inferior fronto-occipital fasciculus as potential biomarkers for resilience [7, 115].

In another study conducted in 65 adolescent females, structural correlates of resilience to depression were reported. Here, resilient females presented with higher functional connectivity between the amygdala and the orbitofrontal cortex, as well as the dorsolateral prefrontal cortex and frontotemporal regions [116]. Based on the reported findings, the authors suggested these functional alterations might enable resilient individuals to better regulate their emotions and behaviors [116]. Taken together, these studies highlight the sex- and gender-specific effects that resilience exerts on both brain and behavior and underscore the necessity to investigate them separately for different sexes and genders [117, 118].

### Context-dependency of risk and protective factors

Protective and risk factors are context-specific and dynamic. Factors that are protective for certain individuals or groups in specific conditions or environments may not be helpful or could even be harmful to others under different conditions or in different environments. Further, the impact of protective factors may fluctuate throughout the lifetime and exert differential effects at different ages.

An example that highlights the intricate relationship between risk and protective factors is the Hispanic Health Paradox or Hispanic Mortality Paradox. It describes the contradictory findings that Hispanic individuals in the United States report better mental and physical health despite lower education and income, which are often considered to be risk factors. Explanations for this span genetic and societal influences that may exert unique effects on this population [119]. These findings may not translate to all domains, as other studies have reported higher levels of stress due to racial and ethnic discrimination and higher levels of depressive and anxiety symptoms during the COVID-19 pandemic in this population [108, 120].

Just as protective factors do not always have a positive impact, risk factors are not always negative. Several studies have identified associations between moderate amounts of stress and more positive health outcomes, such as better coping and less depressive symptoms [121]. This surprising finding has been termed “stress inoculation” and was reported in both animals and humans [122]. The exposure to mildly to moderately stressful events and environments may prepare individuals to deal more effectively with future stressors without overwhelming their capacities [122]. Therefore, exposure to daily stressors, although inconvenient in the short term, may lead to better coping and higher resilience in the long term. We note here that while stress, in specific forms and levels, may be beneficial, we are not suggesting that individuals should be exposed to “small or moderate” amounts of childhood maltreatment or racism. Both experiences are inherently aversive and on the extreme end of the stress spectrum.

Context-dependency has also been observed for a gene-by-environment interaction of a risk and protective factor. A genetic variant of the oxytocin receptor gene associated with increased social sensitivity and higher receptiveness for social support yielded detrimental effects in individuals with a history of childhood maltreatment [123]. In carriers of the higher social sensitivity allele, a dose-dependent effect of exposure to childhood maltreatment was associated with smaller striatal gray matter volume. Based on these findings, the authors concluded that higher social sensitivity was beneficial in positive environments, whereas in adverse environments, it could lead to detrimental effects [89].

Another example of the context-dependency of risk and protective factors is the impact of the conscientiousness personality trait during the COVID-19 pandemic. Conscientiousness is generally considered a protective factor; it entails being organized and responsible and is associated with better academic outcomes and greater life satisfaction [124, 125]. However, during the COVID-19 pandemic, individuals with higher conscientiousness reported higher levels of fear in a study of *N* = 1,268 adults. Since these individuals often benefit from structured and organized environments and situations, the unpredictability of the pandemic may have led to this typically protective trait becoming a risk factor [83]. Similar results were found regarding subjectively reported life satisfaction following loss of employment, where highly conscientious individuals reported greater drops in life satisfaction than other individuals [126]. Therefore, when considering protective and risk factors in research and clinical practice, it is important to recognize their context-specific influences on brain and behavior.

Jointly investigating risk and protective factors also allows for the identification of targeted and effective interventions. A study in 190 Black Americans found that mindfulness successfully buffered against the negative effects of race-related vigilance and was associated with lower levels of depressive symptoms [127]. In a study of 336 (204 female) Asian-American college students, a sex and gender effect was detected in dealing with perceived racism: here, males were more likely to use support-seeking coping strategies, whereas females were more likely to apply active coping strategies. Both are adaptive coping strategies and are considered protective. Surprisingly, both strategies served to actually *increase* racism-related stress in both males and females [128]. These findings illustrate that protective factors are not protective in all settings and may even have opposing effects in specific circumstances. Intervention strategies may need to be tailored to the specific problem. In this instance, individual-focused coping strategies may not suffice in counteracting the adverse effects of a larger, systemic issue such as racism. Moreover, focusing solely on strategies that can be applied at an individual level (e.g., optimism, meditation) unjustly places the burden of action on the already disadvantaged group, rather than addressing the issue at a systemic level. This study further highlights the importance of considering protective factors alongside risk factors, as it can also help identify ineffective strategies, enabling researchers to strategically invest in factors that effectively mitigate adverse psychopathological outcomes.

### Interdependent effects of sex and gender, and risk and protective factors

Our examples underscore the interdependent nature of risk and protective factors. Importantly, interactive effects may even encompass three-way interactions, that include the effect of genetic factors, along with environmental risk and protective factors. Additionally, risk and protective factors impact sex and gender, and vice versa. This joint interplay in turn affects brain and behavior, as illustrated below in Fig. 2. Furthermore, possible feedback loops are conceivable, wherein brain and behavior influence risk and protective factors, and sex and gender. Researchers should be aware of the complex interplay of these factors.

### Beyond binary sex and gender

Important limitations to the generalizability of the findings and recommendations presented here should be noted. First, the discussion in this review is limited to binary sex and gender. Experiences of individuals identifying with other genders might differ as they are exposed to additional stressors that cis-gender individuals may not experience. Few studies have addressed the specific adversities and protective factors that affect transgender and non-binary populations [129–131]. External stressors in these populations include anti-trans legislation and policy, discrimination, violence, and social rejection. Internal stressors include internalized trans-negativity, anticipated stigma, and identity non-disclosure. These additional external and internal stressors can lead to worse mental health outcomes which include substance use, eating disorders, depression, anxiety, and post-traumatic stress disorder [130].

As a drastic example, conversion therapy is the unethical and unscientific attempt to change an individual’s gender identity to fit societal norms. A large study of 7,576 transgender, nonbinary, and gender diverse adolescents and adults in China demonstrated how the practice is associated with significant risk for psychopathology including post-traumatic stress disorder symptoms and suicide attempts [129]. Conversely, gender-affirming medical interventions, legacies, social support, and validating gender identity was found to have a positive impact on nonbinary and transgender individuals in other studies [130–132]. Specifically, validating, actively endorsing, and defending gender-identity along with active learning and self-education emerge as important practices for allies to protect trans and nonbinary individuals’ mental health [131]. Further high-quality research needs to be conducted to systematically elucidate mental health predictors and outcomes in these under-researched populations.

A critical first step will be to acquire relevant sex and gender data in research practice. While this may marginally increase the burden placed on researchers, it will enable us to conduct much-needed research on this topic. Collecting and analyzing data that has previously not been investigated may also lead to new and promising findings.

### Implementation

Currently, there is a mismatch in research such that mental health interventions and psychotherapy focus on improving and strengthening protective factors, but psychiatric research typically does not consider them. In most areas of brain sciences, this has resulted in a focus on risk factors and their influences on mental health. Therefore, a paradigm shift towards a more resource-oriented, rather than deficit-focused approach to mental health is needed. This paradigm-shift can be observed in important research areas of stress resilience, which actively focus on mechanism and effects of adaptive coping with adversity [133–137]. Researchers should be aware of the fact that the absence of a risk factor does not necessarily imply the presence of a protective factors. Some questionnaires may cover risk and protective factors, such as physical exercise or socioeconomic status (where low scores are associated with risk, but high scores are associated with protective effects). However, not all questionnaires span this spectrum, e.g., the absence of childhood maltreatment is not in itself protective. Indeed, a benevolent childhood experiences (BCE) questionnaire was developed as the counterpart to the adverse childhood experiences questionnaire. In a study of 101 pregnant females, BCE were shown to mitigate adverse effects of childhood maltreatment and was associated with lower levels of psychopathology [138]. Therefore, when studying risk factors, researchers should also consider protective measures in their analyses. This may include measures of social support, socioeconomic status, education, resilience, or personality traits. Moreover, these protective measures should not be considered covariates-of-no-interest and regressed out from the analyses. Instead, a statistical approach should be implemented that considers both risk and protective factors, as this allows for the investigation of interaction effects. If data on protective factors is not available, researchers may consider investigating secondary outcomes associated with mental health, such as perceived stress or life satisfaction.

Regarding sex and gender effects, incorporating a dichotomous variable (female/male) is often not sufficient to represent the complexities and lived experiences of sex and gender domains. Economic tools to assess sex and gender include the Diversity Minimal Items Set (DiMIS), which covers nine diversity domains (sex and gender, age, socioeconomic status, care work, sexual orientation, race/ethnicity, religion, disability, and mental health) and their intersections [139]. Ongoing large-scale data collections efforts, such as the Adolescent Brain Cognitive Development study, have also provided information on how to assess sex and gender and related factors in specific populations [140]. These factors should be considered to detect their unique influences on brain, behavior, and mental health rather than “controlling” for sex and gender as a covariate.

In short, researchers should begin to view sex and gender as variables of interest, rather than nuisance variables. They should be precise in the language they use to describe these constructs. Separate analyses should be conducted for different sexes and genders. Researchers should further collect data on gender, which may include questionnaires of gender expression and gender identity. Research should be conducted in diverse populations that include minoritized groups, and non-binary and trans individuals. In interpreting data, researchers should be aware of not perpetuating harmful deficit-focused narratives and take an active anti-sexist stance in their interpretation. For a more detailed discussion on how to successfully account for sex and gender influences in analyses, we refer our readers to [15, 141–143].

## Conclusion

A large focus in current research lies on the investigation of risk factors and their impact on psychopathology and the brain. It is important to identify the factors that most prominently contribute to adverse mental health outcomes. However, these are interconnected and embedded in a multi-dimensional context. Researchers should be aware of the questions we ask, as these shape the narrative around mental health and psychopathology. Focusing on risk factors limits us to insights into the mechanisms that underlie their negative influences on mental wellbeing, perpetuating a deficit-focused view that may, in the worst case, stigmatize individuals with risk factors. The “ordinary magic” of resilience, i.e., the fact that many individuals with risk factors will not develop psychopathology, needs to be acknowledged in both research and clinical practice [144]. Identifying meaningful protective factors for specific population cohorts and understanding their neural correlates and resilience mechanisms will facilitate the development of targeted prevention and intervention programs and improve quality of life for at-risk individuals and individuals with psychiatric illnesses.

The ultimate goal of research in the brain sciences is to provide a comprehensive understanding of the neurobiological mechanisms that underlie behaviors, yielding critical insights into the neural underpinnings of brain disorders. In order for this research to be meaningful, it must be reliable, reproducible, and generalizable. If we do not adequately reflect population diversity – which is associated with significant biological and behavioral diversity – in our research, research findings are likely to be limited in their generalizability. To do this, research needs to include diverse samples especially regarding sex and gender, and race [145]. Investigating these effects does not mean refusing to accept similarities. However, combining these potentially different populations might produce what is called the Simpson paradox: trends in different direction in subgroups might disappear when groups are combined [145]. To avoid this, researchers should perform sex-specific analyses in men and women, and males and females [141].

Sex and gender differences in the prevalence, manifestations, and brain correlates of psychopathology have been repeatedly demonstrated [146, 147]. Therefore, these effects should be considered when researching risk and protective factors for psychiatric disorders. The examples provided here underscore the need to incorporate protective factors into research and investigate sex and gender differences. This will introduce complexity but is also more likely to adequately depict the reality of lived experiences of individuals. Embracing this complexity in research, rather than negating it, will yield better risk prediction and critical findings that may otherwise remain undetected [22].

### Perspectives and significance

Many researchers have advocated for more inclusive practices, and recent advances in this realm show that more inclusive and holistic research is feasible and does not impair the ability to publish [15, 19, 145, 148, 149]. Research is often considered to be objective, but the hypotheses we generate, the samples that we use, and the statistical techniques we apply critically influence the findings we obtain. Focusing entirely on risk factors and their behavioral and brain outcomes will produce more results highlighting the adverse effect of risk factors. Neglecting to investigate men and women, and males and females separately, not including more diverse assessments of sex and gender and not including diverse populations (e.g., trans and non-binary individuals) will generate more research that is blind to sex and gender effects and that supports cisnormativity (i.e., the notion that cis-gender identities are the norm and variation from the gender binary is abnormal) [130]. Therefore, applying novel approaches in research will enable us to change the status quo, reflect the rich diversity in lived experiences, and enable us to acquire deeper findings in research that might in turn be applied to inform meaningful interventions.

## Data Availability

Not applicable.

## References

[1] Nelson J, Klumparendt A, Doebler P, Ehring T. Childhood maltreatment and characteristics of adult depression: Meta-analysis. Br J Psychiatry. 2017;210(2):96–104. 27908895 10.1192/bjp.bp.115.180752

[2] Yang W, Jin S, Duan W, Yu H, Ping L, Shen Z, et al. The effects of childhood maltreatment on cortical thickness and gray matter volume: a coordinate-based meta-analysis. Psychol Med [Internet]. 2023 Mar 22 [cited 2023 Sep 22];53(5). https://pubmed.ncbi.nlm.nih.gov/36946124/10.1017/S0033291723000661 36946124

[3] Goltermann J, Winter NR, Meinert S, Sindermann L, Lemke H, Leehr EJ, et al. Resting-state functional connectivity patterns associated with childhood maltreatment in a large bicentric cohort of adults with and without major depression. Psychol Med [Internet]. 2023 Jul 27 [cited 2023 Sep 27];53(10):4720–31. https://www.cambridge.org/core/journals/psychological-medicine/article/restingstate-functional-connectivity-patterns-associated-with-childhood-maltreatment-in-a-large-bicentric-cohort-of-adults-with-and-without-major-depression/3F9381024994B83AE237739C757D31E6 10.1017/S0033291722001623 PMC10388325 35754405

[4] Ozbay F, Fitterling H, Charney D, Southwick S. Social support and resilience to stress across the life span: a neurobiologic framework. Curr Psychiatry Rep. 2008;10(4):304–10. 18627668 10.1007/s11920-008-0049-7

[5] Struck N, Krug A, Feldmann M, Yuksel D, Stein F, Schmitt S, et al. Attachment and social support mediate the association between childhood maltreatment and depressive symptoms. J Affect Disord. 2020;273(May):310–7. 32421618 10.1016/j.jad.2020.04.041

[6] Pearce M, Garcia L, Abbas A, Strain T, Schuch FB, Golubic R, et al. Association between physical activity and risk of depression: A Systematic Review and Meta-analysis. JAMA Psychiatry [Internet]. 2022 Jun 1 [cited 2023 Feb 23];79(6):550–9. https://jamanetwork.com/journals/jamapsychiatry/fullarticle/2790780 10.1001/jamapsychiatry.2022.0609 PMC9008579 35416941

[7] Brosch K, Stein F, Meller T, Schmitt S, Yuksel D, Ringwald KG, et al. DLPFC volume is a neural correlate of resilience in healthy high-risk individuals with both childhood maltreatment and familial risk for depression. Psychol Med. 2021;1–7. 10.1017/S0033291721001094 PMC9811272 33858550

[8] New AS, Fan J, Murrough JW, Liu X, Liebman RE, Guise KG, et al. A functional magnetic resonance imaging study of deliberate emotion regulation in resilience and posttraumatic stress disorder. Biol Psychiatry [Internet]. 2009;66(7):656–64. 10.1016/j.biopsych.2009.05.020 10.1016/j.biopsych.2009.05.020 19589502

[9] World Health Organisation. https://www.who.int/news-room/fact-sheets/detail/child-maltreatment. 2022. Child Maltreatment.

[10] Antonucci TC, Akiyama H. An examination of sex differences in social support among older men and women. Sex Roles [Internet]. 1987 Dec [cited 2023 Sep 27];17(11–12):737–49. https://link.springer.com/article/10.1007/BF00287685

[11] von Zimmermann C, Hübner M, Mühle C, Müller CP, Weinland C, Kornhuber J, et al. Masculine depression and its problem behaviors: use alcohol and drugs, work hard, and avoid psychiatry! Eur Arch Psychiatry Clin Neurosci [Internet]. 2023 Feb 28 [cited 2023 Sep 25];1:1–13. https://link.springer.com/article/10.1007/s00406-023-01567-0 10.1007/s00406-023-01567-0 PMC10914846 36855002

[12] Pan N, Yang C, Suo X, Shekara A, Hu S, Gong Q, et al. Sex differences in the relationship between brain gray matter volume and psychological resilience in late adolescence. Eur Child Adolesc Psychiatry. 2023 May 22 [cited 2023 May 24];1:1–10. https://link.springer.com/article/10.1007/s00787-023-02231-7 10.1007/s00787-023-02231-7 37212908

[13] Gates Foundation M, Institues of Health N. Women’s Health Innovation Opportunity Map 2023–50 High-return opportunities to advance global women’s health R and D. 2023.

[14] Dhamala E, Bassett DS, Yeo BTT, Holmes AJ. Functional brain networks are associated with both sex and gender in children. Sci Adv [Internet]. 2024 Jul 12 [cited 2024 Jul 17];10(28):4202. https://www.science.org/doi/10.1126/sciadv.adn4202 10.1126/sciadv.adn4202 PMC11244548 38996031

[15] Eliot L, Beery AK, Jacobs EG, LeBlanc HF, Maney DL, McCarthy MM. Why and how to account for sex and gender in brain and behavioral research. J Neurosci. 2023;43(37):6344–56. 37704386 10.1523/JNEUROSCI.0020-23.2023 PMC10500996

[16] Leppert B, Millard LAC, Riglin L, Smith GD, Thapar A, Tilling K, et al. A cross-disorder PRS-pheWAS of 5 major psychiatric disorders in UK Biobank. PLoS Genet [Internet]. 2020 May 1 [cited 2024 Jul 18];16(5):e1008185. https://journals.plos.org/plosgenetics/article?id=10.1371/journal.pgen.1008185 10.1371/journal.pgen.1008185 PMC7274459 32392212

[17] Hess JL, Tylee DS, Mattheisen M, Adolfsson R, Agartz I, Agerbo E, et al. A polygenic resilience score moderates the genetic risk for schizophrenia. Molecular Psychiatry. 2019 26:3 [Internet]. 2019 Sep 6 [cited 2024 Jul 18];26(3):800–15. https://www.nature.com/articles/s41380-019-0463-8 10.1038/s41380-019-0463-8 PMC7058518 31492941

[18] Howard DM, Adams MJ, Clarke TK, Hafferty JD, Gibson J, Shirali M, et al. Genome-wide meta-analysis of depression identifies 102 independent variants and highlights the importance of the prefrontal brain regions. Nat Neurosci [Internet]. 2019;22(3):343–52. 10.1038/s41593-018-0326-7 10.1038/s41593-018-0326-7 PMC6522363 30718901

[19] Jacobs EG. Only 0.5% of neuroscience studies look at women’s health. Here’s how to change that. Nature. 2023;623(7988):667. 37989773 10.1038/d41586-023-03614-1

[20] Johansson T, Vinther Larsen S, Bui M, Ek WE, Karlsson T, Johansson A. Population-based cohort study of oral contraceptive use and risk of depression. Epidemiol Psychiatr Sci [Internet]. 2023 Jun 12 [cited 2024 Jan 16];32:e39. https://www.cambridge.org/core/journals/epidemiology-and-psychiatric-sciences/article/populationbased-cohort-study-of-oral-contraceptive-use-and-risk-of-depression/B3C611DD318D7DC536B4BD439343A5BD 10.1017/S2045796023000525 PMC10294242 37303201

[21] Arciniega GM, Anderson TC, Tovar-Blank ZG, Tracey TJG. Toward a Fuller Conception of Machismo: development of a traditional machismo and Caballerismo Scale. J Couns Psychol. 2008;55(1):19–33.

[22] Cartier L, Guérin M, Saulnier F, Cotocea I, Mohammedi A, Moussaoui F, et al. Sex and gender correlates of sexually polymorphic cognition. Biology of Sex Differences 2024 15:1 [Internet]. 2024 Jan 8 [cited 2024 Jan 23];15(1):1–31. https://bsd.biomedcentral.com/articles/10.1186/s13293-023-00579-8 10.1186/s13293-023-00579-8 PMC10773055 38191503

[23] Mansouri S, Pessoni AM, Marroquín-Rivera A, Parise EM, Tamminga CA, Turecki G et al. Transcriptional dissection of symptomatic profiles across the brain of men and women with depression. Nature Communications 2023 14:1 [Internet]. 2023 Oct 26 [cited 2024 Jul 29];14(1):1–14. https://www.nature.com/articles/s41467-023-42686-5 10.1038/s41467-023-42686-5 PMC10603117 37884562

[24] Parrish E. The next pandemic: COVID-19 mental health pandemic. Perspectives in Psychiatric Care. 2020. 10.1111/ppc.12571 32602165

[25] Fitzpatrick KM, Harris C, Drawve G. Fear of COVID-19 and the Mental Health Consequences in America. Psychol Trauma. 2020;12:17–21. 10.1037/tra0000924 32496100

[26] Madigan S, Deneault AA, Racine N, Park J, Thiemann R, Zhu J, et al. Adverse childhood experiences: a meta-analysis of prevalence and moderators among half a million adults in 206 studies. World Psychiatry [Internet]. 2023 Oct 1 [cited 2024 Jan 10];22(3):463–71. https://onlinelibrary.wiley.com/doi/full/10.1002/wps.21122 10.1002/wps.21122 PMC10503911 37713544

[27] Pierson E. Accuracy and Equity in Clinical Risk Prediction. https://doi.org/101056/NEJMp2311050 [Internet]. 2024 Jan 6 [cited 2024 Jan 11];390(2):100–2. https://www.nejm.org/doi/full/10.1056/NEJMp2311050 10.1056/NEJMp2311050 38198167

[28] Wang F, Gao Y, Han Z, Yu Y, Long Z, Jiang X, et al. A systematic review and meta-analysis of 90 cohort studies of social isolation, loneliness and mortality. Nature Human Behaviour 2023 7:8 [Internet]. 2023 Jun 19 [cited 2024 Jan 11];7(8):1307–19. https://www.nature.com/articles/s41562-023-01617-6 10.1038/s41562-023-01617-6 37337095

[29] Holt-Lunstad J, Smith TB, Baker M, Harris T, Stephenson D. Loneliness and social isolation as risk factors for mortality: a Meta-Analytic Review. Perspect Psychol Sci. 2015;10(2):227–37. 25910392 10.1177/1745691614568352

[30] Flory JD, Yehuda R, Passarelli V, Siever LJ. Joint Effect of Childhood Abuse and Family History of Major Depressive Disorder on Rates of PTSD in People with Personality Disorders. Vermetten E, editor. Depress Res Treat [Internet]. 2012;2012:350461. 10.1155/2012/350461 10.1155/2012/350461 PMC3335173 22577531

[31] Navrady LB, Adams MJ, Chan SWY, Consortium MDDWG, of the SJ, McIntosh AM. Genetic risk of major depressive disorder: the moderating and mediating effects of neuroticism and psychological resilience on clinical and self-reported depression. Psychol Med [Internet]. 2017;1–10. http://files/824/genetic_risk_of_major_depressive_disorder_the_moderating_and_mediating_effects_of_neuroticism_and_psychological_resilience_on_clinical_and_selfreported_depression.pdf%0Ahttp://www.ncbi.nlm.nih.gov/pubmed/29183409 10.1017/S0033291717003415 PMC6088772 29183409

[32] Walker EA, Gelfand A, Katon WJ, Koss MP, Von Korff M, Bernstein D, et al. Adult health status of women with histories of childhood abuse and neglect. Am J Med. 1999;107(4):332–9. 10527034 10.1016/s0002-9343(99)00235-1

[33] Heim CM, Mayberg HS, Mletzko T, Nemeroff CB, Pruessner JC. Decreased cortical representation of genital somatosensory field after childhood sexual abuse. Am J Psychiatry. 2013;170(6):616–23. 23732967 10.1176/appi.ajp.2013.12070950

[34] Tomoda A, Navalta CP, Polcari A, Sadato N, Teicher MH. Childhood Sexual Abuse Is Associated with Reduced Gray Matter Volume in Visual Cortex of Young Women. Biol Psychiatry [Internet]. 2009;66(7):642–8. 10.1016/j.biopsych.2009.04.021 10.1016/j.biopsych.2009.04.021 PMC4277202 19560122

[35] Nanni V, Uher R, Danese A. Childhood maltreatment predicts unfavorable course of illness and treatment outcome in depression: a meta-analysis. Am J Psychiatry. 2012;169(2):141–51. 22420036 10.1176/appi.ajp.2011.11020335

[36] Gallo EAG, Munhoz TN, Loret de Mola C, Murray J. Gender differences in the effects of childhood maltreatment on adult depression and anxiety: a systematic review and meta-analysis. Child Abuse Negl. 2018;79(March 2017):107–14. 29428878 10.1016/j.chiabu.2018.01.003

[37] Hailes HP, Yu R, Danese A, Fazel S. Long-term outcomes of childhood sexual abuse: an umbrella review. Lancet Psychiatry. 2019;6(10):830–9. 31519507 10.1016/S2215-0366(19)30286-X PMC7015702

[38] Baldwin JR, Wang B, Karwatowska L, Schoeler T, Tsaligopoulou A, Munafò MR et al. Childhood Maltreatment and Mental Health Problems: A Systematic Review and Meta-Analysis of Quasi-Experimental Studies. https://doi.org/101176/appi.ajp20220174 [Internet]. 2023 Jan 11 [cited 2024 Jan 10];180(2):117–26. 10.1176/appi.ajp.20220174 10.1176/appi.ajp.20220174 PMC7614155 36628513

[39] Opel N, Zwanzger P, Redlich R, Grotegerd D, Dohm K, Arolt V, et al. Differing brain structural correlates of familial and environmental risk for major depressive disorder revealed by a combined VBM/pattern recognition approach. Psychol Med. 2016;46(2):277–90. 26355299 10.1017/S0033291715001683

[40] Gerin MI, Viding E, Herringa RJ, Russell JD, Mccrory EJ. A systematic review of childhood maltreatment and resting state functional connectivity. Dev Cogn Neurosci [Internet]. 2023 [cited 2024 Jan 1];64:101322. 10.1016/j.dcn.2023.101322 10.1016/j.dcn.2023.101322 PMC10665826 37952287

[41] Teicher MH, Samson JA, Anderson CM, Ohashi K. The effects of childhood maltreatment on brain structure, function and connectivity. Nat Rev Neurosci [Internet]. 2016;17(10):652–66. 10.1038/nrn.2016.111 10.1038/nrn.2016.111 27640984

[42] Khan A, McCormack HC, Bolger EA, McGreenery CE, Vitaliano G, Polcari A, et al. Childhood maltreatment, depression, and suicidal ideation: Critical importance of parental and peer emotional abuse during developmental sensitive periods in males and females. Front Psychiatry. 2015;6(MAR):132663. 10.3389/fpsyt.2015.00042 PMC4378368 25870565

[43] Millman ZB, Hwang M, Sydnor VJ, Reid BE, Goldenberg JE, Talero JN, et al. Auditory hallucinations, childhood sexual abuse, and limbic gray matter volume in a transdiagnostic sample of people with psychosis. Schizophrenia 2022 8:1 [Internet]. 2022 Dec 30 [cited 2024 Jan 1];8(1):1–9. https://www.nature.com/articles/s41537-022-00323-y 10.1038/s41537-022-00323-y PMC9803640 36585407

[44] Paquola C, Bennett MR, Lagopoulos J. Understanding heterogeneity in grey matter research of adults with childhood maltreatment—A meta-analysis and review. Neurosci Biobehav Rev [Internet]. 2016;69:299–312. 10.1016/j.neubiorev.2016.08.011 10.1016/j.neubiorev.2016.08.011 27531235

[45] Tozzi L, Garczarek L, Janowitz D, Stein DJ, Wittfeld K, Dobrowolny H, et al. Interactive impact of childhood maltreatment, depression, and age on cortical brain structure: mega-analytic findings from a large multi-site cohort. Psychol Med. 2020;50(6):1020–31. 31084657 10.1017/S003329171900093X PMC9254722

[46] Kendler KS, Karkowski LM, Prescott CA. Causal relationship between stressful life events and the onset of major depression. Am J Psychiatry. 1999;156(6):837–41. 10360120 10.1176/ajp.156.6.837

[47] Douaud G, Lee S, Alfaro-Almagro F, Arthofer C, Wang C, McCarthy P, et al. SARS-CoV-2 is associated with changes in brain structure in UK Biobank. Nature 2022 604:7907 [Internet]. 2022 Mar 7 [cited 2023 Oct 23];604(7907):697–707. https://www.nature.com/articles/s41586-022-04569-5 10.1038/s41586-022-04569-5 PMC9046077 35255491

[48] Hatzenbuehler ML, McLaughlin KA, Weissman DG, Cikara M. A research agenda for understanding how social inequality is linked to brain structure and function. Nature Human Behaviour 2024 [Internet]. 2024 Jan 3 [cited 2024 Jan 18];1–12. https://www.nature.com/articles/s41562-023-01774-8 10.1038/s41562-023-01774-8 PMC11112523 38172629

[49] Ricard JA, Parker TC, Dhamala E, Kwasa J, Allsop A, Holmes AJ. Confronting racially exclusionary practices in the acquisition and analyses of neuroimaging data. Nature Neuroscience. 2022 26:1 [Internet]. 2022 Dec 23 [cited 2024 Jan 18];26(1):4–11. https://www.nature.com/articles/s41593-022-01218-y 10.1038/s41593-022-01218-y PMC12884511 36564545

[50] Mach KJ, Salas Reyes R, Pentz B, Taylor J, Costa CA, Cruz SG, et al. News media coverage of COVID-19 public health and policy information. Humanities and Social Sciences Communications 2021 8:1 [Internet]. 2021 Sep 28 [cited 2024 Jan 18];8(1):1–11. https://www.nature.com/articles/s41599-021-00900-z

[51] Nobles M, Womack C, Wonkam A, Wathuti E. Science must overcome its racist legacy: Nature’s guest editors speak. Nature. 2022;606(7913):225–7. 35676434 10.1038/d41586-022-01527-z

[52] Müller R, Ruess AK, Schönweitz FB, Buyx A, Gil Ávila C, Ploner M. Next steps for global collaboration to minimize racial and ethnic bias in neuroscience. Nature Neuroscience 2023 26:7 [Internet]. 2023 Jun 9 [cited 2024 Jan 18];26(7):1132–3. https://www.nature.com/articles/s41593-023-01369-6 10.1038/s41593-023-01369-6 37296224

[53] Brosch K, Stein F, Schmitt S, Pfarr JK, Ringwald KG, Thomas-Odenthal F, et al. Reduced hippocampal gray matter volume is a common feature of patients with major depression, bipolar disorder, and schizophrenia spectrum disorders. Mol Psychiatry [Internet]. 2022;i(June):1–10. http://www.ncbi.nlm.nih.gov/pubmed/35840798 10.1038/s41380-022-01687-4 PMC9718668 35840798

[54] Ringwald KG, Meller T, Schmitt S, Andlauer TFM, Stein F, Brosch K, et al. Interaction of developmental factors and ordinary stressful life events on brain structure in adults. Neuroimage Clin. 2021;30. 10.1016/j.nicl.2021.102683 PMC8102615 34215153

[55] Ringwald KG, Pfarr JK, Stein F, Brosch K, Meller T, Thomas-Odenthal F, et al. Association between stressful life events and grey matter volume in the medial prefrontal cortex: A 2-year longitudinal study. Hum Brain Mapp [Internet]. 2022 Aug 1 [cited 2023 Dec 18];43(11):3577–84. https://pubmed.ncbi.nlm.nih.gov/35411559/10.1002/hbm.25869 PMC9248310 35411559

[56] Hosseini-Kamkar N, Varvani Farahani M, Nikolic M, Stewart K, Goldsmith S, Soltaninejad M, et al. Adverse Life Experiences and Brain Function: A Meta-Analysis of Functional Magnetic Resonance Imaging Findings. JAMA Netw Open [Internet]. 2023 Nov 1 [cited 2024 Feb 11];6(11):e2340018–e2340018. https://jamanetwork.com/journals/jamanetworkopen/fullarticle/2811174 10.1001/jamanetworkopen.2023.40018 PMC10620621 37910106

[57] Kendler KS, Thornton LM, Prescott CA. Gender differences in the rates of exposure to stressful life events and sensitivity to their depressogenic effects. American Journal of Psychiatry [Internet]. 2001 Apr 1 [cited 2023 Dec 18];158(4):587–93. 10.1176/appi.ajp.158.4.587 10.1176/appi.ajp.158.4.587 11282693

[58] Paradies Y, Ben J, Denson N, Elias A, Priest N, Pieterse A, et al. Racism as a Determinant of Health: A Systematic Review and Meta-Analysis. PLoS One [Internet]. 2015 Sep 23 [cited 2024 Jan 30];10(9):e0138511. https://journals.plos.org/plosone/article?id=10.1371/journal.pone.0138511 10.1371/journal.pone.0138511 PMC4580597 26398658

[59] Torres-Harding S, Turner T. Assessing Racial Microaggression Distress in a Diverse Sample. http://dx.doi.org/10.1177/0163278714550860 [Internet]. 2014 Sep 18 [cited 2024 Jul 7];38(4):464–90. https://journals.sagepub.com/doi/full/10.1177/0163278714550860 10.1177/0163278714550860 25237154

[60] Dumornay NM, Lebois LAM, Ressler KJ, Harnett NG. Racial Disparities in Adversity During Childhood and the False Appearance of Race-Related Differences in Brain Structure. American Journal of Psychiatry [Internet]. 2023 Feb 1 [cited 2023 Dec 4];180(2):127–38. 10.1176/appi.ajp.21090961 10.1176/appi.ajp.21090961 PMC9897449 36722118

[61] Harnett NG, Wheelock MD, Wood KH, Goodman AM, Mrug S, Elliott MN, et al. Negative life experiences contribute to racial differences in the neural response to threat. NeuroImage. 2019;202:116086. 31401241 10.1016/j.neuroimage.2019.116086 PMC6819267

[62] Muscatell KA, Alvarez GM, Bonar AS, Cardenas MN, Galvan MJ, Merritt CC, et al. Brain–Body Pathways Link Racism Health Am Psychol. 2022;77(9):1049–60. 10.1037/amp0001084 PMC9887645 36595402

[63] Harrell CJP, Burford TI, Cage BN, Nelson TM, Shearon S, Thompson A et al. Multiple Pathways Linking Racism to Health Outcomes. Du Bois Rev [Internet]. 2011 Apr 4 [cited 2024 Jul 8];8(1):143. http://pmc/articles/PMC3328094/10.1017/S1742058X11000178 PMC3328094 22518195

[64] Fulton T, Lathan EC, Karkare MC, Guelfo A, Eghbalzad L, Ahluwalia V, et al. Civilian Moral Injury and Amygdala Functional Connectivity during attention to threat. Biol Psychiatry Cogn Neurosci Neuroimaging. 2024;9(1):112–20. 37487958 10.1016/j.bpsc.2023.07.006 PMC10803642

[65] Harnett NG. The moderating role of Moral Injury on the neurocircuitry of trauma-related dysfunction. Biol Psychiatry Cogn Neurosci Neuroimaging. 2024;9(1):6–7. 38185486 10.1016/j.bpsc.2023.11.005

[66] Michaels TI, Thomas E, Flaxer JM, Singal S, Hanna L, Van Meter A, et al. Racial and ethnic inequities in psychiatric inpatient building and unit assignment. Psychiatry Res. 2023;330:115560. 37956588 10.1016/j.psychres.2023.115560

[67] Causadias JM, Korous KM. Racial Discrimination in the United States: A National Health Crisis That Demands a National Health Solution. Journal of Adolescent Health [Internet]. 2019 Feb 1 [cited 2024 Jul 9];64(2):147–8. http://www.jahonline.org/article/S1054139X18307845/fulltext 10.1016/j.jadohealth.2018.11.001 30660246

[68] Nagata JM, Ganson KT, Sajjad OM, Benabou SE, Bibbins-Domingo K. Prevalence of Perceived Racism and Discrimination Among US Children Aged 10 and 11 Years: The Adolescent Brain Cognitive Development (ABCD) Study. JAMA Pediatr [Internet]. 2021 Aug 1 [cited 2024 Mar 12];175(8):861. http://pmc/articles/PMC8129899/10.1001/jamapediatrics.2021.1022 PMC8129899 33999104

[69] Cardenas-Iniguez C, Gonzalez MR. Recommendations for the responsible use and communication of race and ethnicity in neuroimaging research. Nature Neuroscience 2024 [Internet]. 2024 Mar 22 [cited 2024 Mar 26];1–14. https://www.nature.com/articles/s41593-024-01608-4 10.1038/s41593-024-01608-4 PMC11698468 38519749

[70] Epstein R, Blake J, González T. Girlhood Interrupted: The Erasure of Black Girls’ Childhood. SSRN Electronic Journal [Internet]. 2017 Jun 27 [cited 2024 Jan 29]; https://papers.ssrn.com/abstract=3000695

[71] Perillo JT, Sykes RB, Bennett SA, Reardon MC. Examining the consequences of Dehumanization and Adultification in Justification of Police Use of Force Against Black Girls and boys. Law Hum Behav. 2023;47(1):36–52. 36931848 10.1037/lhb0000521

[72] Nuamah SA, Mulroy Q. I am a Child! Public Perceptions of Black Girls and their Punitive Consequences. J Race Ethn Polit [Internet]. 2023 Jul 21 [cited 2024 Jan 29];8(2):182–201. https://www.cambridge.org/core/journals/journal-of-race-ethnicity-and-politics/article/abs/i-am-a-child-public-perceptions-of-black-girls-and-their-punitive-consequences/B35A411A3E19B65E3ABB316890784967

[73] Goff PA, Jackson MC, Di Leone BAL, Culotta CM, DiTomasso NA. The essence of innocence: consequences of dehumanizing black children. J Pers Soc Psychol. 2014;106(4):526–45. 24564373 10.1037/a0035663

[74] Berger M, Sarnyai Z. More than skin deep: stress neurobiology and mental health consequences of racial discrimination. Stress [Internet]. 2015 Jan 1 [cited 2024 Jan 16];18(1):1–10. https://www.tandfonline.com/doi/abs/10.3109/10253890.2014.989204 10.3109/10253890.2014.989204 25407297

[75] Rosette AS, Ponce de Leon R, Koval CZ, Harrison DA. Intersectionality: connecting experiences of gender with race at work. Res Organ Behav. 2018;38:1–22.

[76] Okeke O, Elbasheir A, Carter SE, Powers A, Mekawi Y, Gillespie CF, et al. Indirect effects of racial discrimination on Health outcomes through prefrontal cortical White Matter Integrity. Biol Psychiatry Cogn Neurosci Neuroimaging. 2023;8(7):741–9. 35597432 10.1016/j.bpsc.2022.05.004

[77] Fani N, Eghbalzad L, Harnett NG, Carter SE, Price M, Stevens JS, et al. Racial discrimination associates with lower cingulate cortex thickness in trauma-exposed black women. Neuropsychopharmacology 2022 47:13 [Internet]. 2022 Sep 13 [cited 2024 Jan 30];47(13):2230–7. https://www.nature.com/articles/s41386-022-01445-8 10.1038/s41386-022-01445-8 PMC9630426 36100659

[78] Davis HE, McCorkell L, Vogel JM, Topol EJ, Long. COVID: major findings, mechanisms and recommendations. Nature Reviews Microbiology 2023 [Internet]. 2023 Jan 13 [cited 2023 Jan 16];1–14. https://www.nature.com/articles/s41579-022-00846-2 10.1038/s41579-022-00846-2 PMC9839201 36639608

[79] Chen C, Haupert SR, Zimmermann L, Shi X, Fritsche LG, Mukherjee B. Global Prevalence of Post-Coronavirus Disease 2019 (COVID-19) condition or long COVID: a meta-analysis and systematic review. J Infect Dis [Internet]. 2022 Nov 1 [cited 2024 Jan 2];226(9):1593–607. 10.1093/infdis/jiac136 10.1093/infdis/jiac136 PMC9047189 35429399

[80] Monje M, Iwasaki A. The neurobiology of long COVID. Neuron [Internet]. 2022 [cited 2023 Jan 17];110:3484–96. 10.1016/j.neuron.2022.10.006 10.1016/j.neuron.2022.10.006 PMC9537254 36288726

[81] Díez-Cirarda M, Yus M, Gómez-Ruiz N, Polidura C, Gil-Martínez L, Delgado-Alonso C, et al. Multimodal neuroimaging in post-COVID syndrome and correlation with cognition. Brain [Internet]. 2023 May 1 [cited 2023 Oct 23];146(5):2142–52. Available from: /pmc/articles/PMC9620345/. 10.1093/brain/awac384 PMC9620345 36288544

[82] Flor LS, Friedman J, Spencer CN, Cagney J, Arrieta A, Herbert ME, et al. Quantifying the effects of the COVID-19 pandemic on gender equality on health, social, and economic indicators: a comprehensive review of data from March, 2020, to September, 2021. The Lancet [Internet]. 2022 Jun 25 [cited 2023 Dec 18];399(10344):2381–97. http://www.thelancet.com/article/S0140673622000083/fulltext 10.1016/S0140-6736(22)00008-3 PMC8890763 35247311

[83] Brosch K, Meller T, Pfarr JK, Stein F, Schmitt S, Ringwald KG, et al. Which traits predict elevated distress during the Covid-19 pandemic? Results from a large, longitudinal cohort study with psychiatric patients and healthy controls. J Affect Disord. 2022;297(September 2021):18–25. 34670129 10.1016/j.jad.2021.10.017 PMC8520504

[84] Luo M, Guo L, Yu M, Wang H. The psychological and mental impact of coronavirus disease 2019 (COVID-19) on medical staff and general public – a systematic review and meta-analysis. Psychiatry Res. 2020. 10.1016/j.psychres.2020.113190 PMC7276119 32563745

[85] Vindegaard N, Benros ME. COVID-19 pandemic and mental health consequences: systematic review of the current evidence. Behavior, and Immunity: Brain; 2020. 10.1016/j.bbi.2020.05.048 PMC7260522 32485289

[86] Xiong J, Lipsitz O, Nasri F, Lui LMW, Gill H, Phan L, et al. Impact of COVID-19 pandemic on mental health in the general population: a systematic review. J Affect Disord. 2020. 10.1016/j.jad.2020.08.001 PMC7413844 32799105

[87] Masten AS, Lucke CM, Nelson KM, Stallworthy IC. Resilience in Development and psychopathology: multisystem perspectives. Annu Rev Clin Psychol. 2021;17:521–49. 33534615 10.1146/annurev-clinpsy-081219-120307

[88] Verhoeven JE, Han LKM, Lever-van Milligen BA, Hu MX, Révész D, Hoogendoorn AW, et al. Antidepressants or running therapy: comparing effects on mental and physical health in patients with depression and anxiety disorders. J Affect Disord. 2023;329:19–29. 36828150 10.1016/j.jad.2023.02.064

[89] Tian YE, Di Biase MA, Mosley PE, Lupton MK, Xia Y, Fripp J, et al. Evaluation of Brain-Body Health in Individuals With Common Neuropsychiatric Disorders. JAMA Psychiatry [Internet]. 2023 Jun 1 [cited 2024 Jan 18];80(6):567–76. https://jamanetwork.com/journals/jamapsychiatry/fullarticle/2804355 10.1001/jamapsychiatry.2023.0791 PMC10134046 37099313

[90] Shahyad S, Besharat MA, Asadi M, ShirAlipour A, Miri M. The Relation of Attachment and perceived social support with Life Satisfaction: Structural Equation Model. In: Procedia - Social and Behavioral Sciences. 2011.

[91] Ong AD, Bergeman CS, Bisconti TL, Wallace KA. Psychological resilience, positive emotions, and successful adaptation to stress in later life. J Pers Soc Psychol. 2006;91(4):730–49. 17014296 10.1037/0022-3514.91.4.730

[92] Ozbay F, Johnson DC, Dimoulas E, Morgan CA, Charney I, Southwick D. S. Social Support and Resilience to Stress: From Neurobiology to Clinical Practice. Psychiatry (Edgmont) [Internet]. 2007 May [cited 2024 Jan 18];4(5):35. http://pmc/articles/PMC2921311/PMC2921311 20806028

[93] Vila J. Social Support and Longevity: Meta-Analysis-based evidence and psychobiological mechanisms. Front Psychol [Internet]. 2021 Sep 13 [cited 2024 Jul 18];12:717164. Available from: www.frontiersin.org. 10.3389/fpsyg.2021.717164 PMC8473615 34589025

[94] Flinkenflügel K, Meinert S, Thiel K, Winter A, Goltermann J, Strathausen L, et al. Negative Stressful Life Events and Social Support Are Associated With White Matter Integrity in Depressed Patients and Healthy Control Participants: A Diffusion Tensor Imaging Study. Biol Psychiatry [Internet]. 2023 Oct 15 [cited 2024 Jan 4];94(8):650–60. https://pubmed.ncbi.nlm.nih.gov/37028741/10.1016/j.biopsych.2023.03.022 37028741

[95] Che XW, Wei DT, Li WF, Li HJ, Qiao L, Qiu J, et al. The correlation between gray matter volume and perceived social support: a voxel-based morphometry study. Soc Neurosci. 2014;9(2). 10.1080/17470919.2013.873078 24397344

[96] Hu T, Zhang D, Wang J. A meta-analysis of the trait resilience and mental health. Pers Individ Dif [Internet]. 2015;76:18–27. 10.1016/j.paid.2014.11.039

[97] Wagnild GM, Young HM. Development and psychometric evaluation of the Resilience Scale. J Nurs Meas. 1993;1:165–78. 7850498

[98] Smith BW, Dalen J, Wiggins K, Tooley E, Christopher P, Bernard J. The brief resilience scale: assessing the ability to bounce back. Int J Behav Med. 2008;15(3):194–200. 18696313 10.1080/10705500802222972

[99] Campbell-Sills L, Stein MB. Psychometric analysis and refinement of the Connor-Davidson Resilience Scale (CD-RISC): validation of a 10-item measure of resilience. J Trauma Stress. 2007;20(6). 10.1002/jts.20271 18157881

[100] Freund PA, Kasten N. How smart do you think you are? A meta-analysis on the validity of self-estimates of cognitive ability. Psychol Bull. 2012;138(2):296–321. 22181852 10.1037/a0026556

[101] Kahl M, Wagner G, de la Cruz F, Köhler S, Schultz CC. Resilience and cortical thickness: a MRI study. Eur Arch Psychiatry Clin Neurosci [Internet]. 2018;0(0):0. 10.1007/s00406-018-0963-6 10.1007/s00406-018-0963-6 30542819

[102] Ioannidis K, Askelund AD, Kievit RA, Van Harmelen AL. The complex neurobiology of resilient functioning after childhood maltreatment. BMC Med. 2020. 10.1186/s12916-020-1490-7 PMC7017563 32050974

[103] Buchanan M, Walker G, Boden JM, Mansoor Z, Newton-Howes G. Protective factors for psychosocial outcomes following cumulative childhood adversity: systematic review. BJPsych Open [Internet]. 2023 Nov 19 [cited 2023 Oct 24];9(6):e197. https://www.cambridge.org/core/journals/bjpsych-open/article/protective-factors-for-psychosocial-outcomes-following-cumulative-childhood-adversity-systematic-review/03C2C8E7B024392F3D40FB85A59C470A 10.1192/bjo.2023.561 PMC10594245 37855106

[104] Vanbronkhorst SB, Abraham E, Dambreville R, Ramos-Olazagasti MA, Wall M, Saunders DC et al. Sociocultural Risk and Resilience in the Context of Adverse Childhood Experiences. JAMA Psychiatry [Internet]. 2023 Dec 27 [cited 2024 Jan 3]; https://jamanetwork.com/journals/jamapsychiatry/fullarticle/2813435 10.1001/jamapsychiatry.2023.4900 PMC10753442 38150238

[105] Choi KW, Lee YH, Liu Z, Fatori D, Bauermeister JR, Luh RA et al. Social support and depression during a global crisis. Nature Mental Health 2023 1:6 [Internet]. 2023 Jun 8 [cited 2024 Jan 10];1(6):428–35. https://www.nature.com/articles/s44220-023-00078-0

[106] Hayes AF, Rockwood NJ. Regression-based statistical mediation and moderation analysis in clinical research: observations, recommendations, and implementation. Behav Res Ther. 2017;98. 10.1016/j.brat.2016.11.001 27865431

[107] Förster K, Danzer L, Redlich R, Opel N, Grotegerd D, Leehr EJ et al. Social support and hippocampal volume are negatively associated in adults with previous experience of childhood maltreatment. J Psychiatry Neurosci. 2021;46(3). 10.1503/jpn.200162 PMC8327979 33904668

[108] Rahman A, Sánchez M, Bursac Z, Whiting CY, de Dios MA, Cano M, et al. Ethnic discrimination and psychological stress among hispanic emerging adults: examining the moderating effects of distress tolerance and optimism. Int J Intercultural Relations. 2022;86:217–26. 10.1016/j.ijintrel.2021.12.005 PMC9540438 36212111

[109] Adkins-Jackson PB, Turner-Musa J, Chester C. The Path to Better Health for Black Women: Predicting Self-Care and Exploring Its Mediating Effects on Stress and Health. Inquiry (United States) [Internet]. 2019 Sep 5 [cited 2024 Jan 17];56. https://journals.sagepub.com/doi/10.1177/0046958019870968 10.1177/0046958019870968 PMC6728668 31486346

[110] Grollman EA. Multiple forms of perceived discrimination and health among adolescents and young adults. J Health Soc Behav [Internet]. 2012 Jun [cited 2024 Jan 16];53(2):199–214. https://pubmed.ncbi.nlm.nih.gov/22588219/10.1177/0022146512444289 22588219

[111] Grollman EA. Multiple Forms of Perceived Discrimination and Health among Adolescents and Young Adults. https://doi.org/101177/0022146512444289 [Internet]. 2012 May 15 [cited 2024 Jan 16];53(2):199–214. https://journals.sagepub.com/doi/10.1177/0022146512444289?url_ver=Z39.88-2003&rfr_id=ori%3Arid%3Acrossref.org&rfr_dat=cr_pub++0pubmed 10.1177/0022146512444289 22588219

[112] Yip SW, Jordan A, Kohler RJ, Holmes A, Bzdok D, Multivariate. Transgenerational Associations of the COVID-19 Pandemic Across Minoritized and Marginalized Communities. JAMA Psychiatry [Internet]. 2022 Apr 1 [cited 2024 Jan 30];79(4):350–8. https://jamanetwork.com/journals/jamapsychiatry/fullarticle/2788897 10.1001/jamapsychiatry.2021.4331 PMC8829750 35138333

[113] Kalisch R, Cramer AOJ, Binder H, Fritz J, Leertouwer Ij, Lunansky G, et al. Deconstructing and reconstructing resilience: a Dynamic Network Approach. Perspectives on Psychological Science; 2019. 10.1177/1745691619855637 31365841

[114] Chmitorz A, Kunzler A, Helmreich I, Tüscher O, Kalisch R, Kubiak T et al. Intervention studies to foster resilience – A systematic review and proposal for a resilience framework in future intervention studies. Clin Psychol Rev [Internet]. 2018;59(January 2017):78–100. 10.1016/j.cpr.2017.11.002 10.1016/j.cpr.2017.11.002 29167029

[115] Winter A, Thiel K, Meinert S, Lemke H, Waltemate L, Breuer F et al. Familial risk for major depression: differential white matter alterations in healthy and depressed participants. Psychol Med [Internet]. 2023 Aug 2 [cited 2024 Jan 4];53(11):4933–42. https://pubmed.ncbi.nlm.nih.gov/36052484/10.1017/S003329172200188X PMC10476061 36052484

[116] Fischer AS, Camacho MC, Ho TC, Whitfield-Gabrieli S, Gotlib IH. Neural markers of resilience in adolescent females at familial risk for major depressive disorder. JAMA Psychiatry. 2018;75(5):493–502. 29562053 10.1001/jamapsychiatry.2017.4516 PMC5875355

[117] Bolsinger J, Seifritz E, Kleim B, Manoliu A. Neuroimaging correlates of resilience to traumatic Events—A Comprehensive Review. Front Psychiatry. 2018;9:383132. 10.3389/fpsyt.2018.00693 PMC6315158 30631288

[118] Afifi TO, MacMillan HL. Resilience following child maltreatment: a review of protective factors. Can J Psychiatry [Internet]. 2011 [cited 2023 Jul 25];56(5):266–72. https://pubmed.ncbi.nlm.nih.gov/21586192/10.1177/070674371105600505 21586192

[119] Fernandez J, García-Pérez M, Orozco-Aleman S. Unraveling the Hispanic Health Paradox. Journal of Economic Perspectives [Internet]. 2023 [cited 2024 Jan 16];37(1):145–68. 10.1257/jep.37.1.145

[120] Jamil B, Su J. Multidimensional social support and associations between COVID-19 stress and depressive/anxiety outcomes among Hispanic/Latinx and White first-year college students. Journal of American College Health [Internet]. 2024 Jan 16 [cited 2024 Jan 17];1–12. https://www.tandfonline.com/doi/full/10.1080/07448481.2023.2299413 10.1080/07448481.2023.2299413 38227914

[121] Shapero BG, Hamilton JL, Stange JP, Liu RT, Abramson LY, Alloy LB. Moderate Childhood Stress Buffers Against Depressive Response to Proximal Stressors: A Multi-Wave Prospective Study of Early Adolescents. J Abnorm Child Psychol [Internet]. 2015 Nov 1 [cited 2024 Jan 17];43(8):1403. http://pmc/articles/PMC4609240/10.1007/s10802-015-0021-z PMC4609240 25911194

[122] Ayash S, Schmitt U, Lyons DM, Müller MB. Stress inoculation in mice induces global resilience. Translational Psychiatry. 2020 10:1 [Internet]. 2020 Jun 19 [cited 2024 Jan 17];10(1):1–8. https://www.nature.com/articles/s41398-020-00889-0 10.1038/s41398-020-00889-0 PMC7305209 32561821

[123] Dannlowski U, Kugel H, Grotegerd D, Redlich R, Opel N, Dohm K et al. Disadvantage of Social Sensitivity: Interaction of Oxytocin Receptor Genotype and Child Maltreatment on Brain Structure. Biol Psychiatry [Internet]. 2016 Sep 1 [cited 2023 Dec 4];80(5):398–405. https://pubmed.ncbi.nlm.nih.gov/26858213/10.1016/j.biopsych.2015.12.010 26858213

[124] Malouff JM, Thorsteinsson EB, Schutte NS. The relationship between the five-factor model of personality and symptoms of clinical disorders: a meta-analysis. J Psychopathol Behav Assess. 2005.

[125] Hayes N, Joseph S. Big 5 correlates of three measures of subjective well-being. Pers Individ Dif. 2003.

[126] Boyce CJ, Wood AM, Brown GDA. The dark side of conscientiousness: conscientious people experience greater drops in life satisfaction following unemployment. J Res Pers. 2010;44(4).

[127] Watson-Singleton NN, Hill LBK, Case AD, Past Discrimination R-R, Vigilance. and Depressive Symptoms: the Moderating Role of Mindfulness. Mindfulness (N Y) [Internet]. 2019 Sep 1 [cited 2024 Jul 9];10(9):1768–78. https://link.springer.com/article/10.1007/s12671-019-01143-5 10.1007/s12671-019-01143-5 PMC6892459 31803305

[128] Liang CTH, Alvarez AN, Juang LP, Liang MX. The role of coping in the Relationship between Perceived Racism and Racism-related stress for Asian americans: gender differences. J Couns Psychol. 2007;54(2).

[129] Wang Y, Han M, Zhang Y, Wang Y, Li G, Huang Z et al. A national transgender health survey from China assessing gender identity conversion practice, mental health, substance use and suicidality. Nature Mental Health 2023 1:4 [Internet]. 2023 Apr 18 [cited 2024 Jan 10];1(4):254–65. https://www.nature.com/articles/s44220-023-00041-z

[130] Tebbe EA, Budge SL. Factors that drive mental health disparities and promote well-being in transgender and nonbinary people. Nature Reviews Psychology 2022 1:12 [Internet]. 2022 Sep 26 [cited 2024 Jan 10];1(12):694–707. https://www.nature.com/articles/s44159-022-00109-0 10.1038/s44159-022-00109-0 PMC9513020 36187743

[131] Galupo MP, Pulice-Farrow L, Clements ZA, Morris ER. I love you as both and I love you as neither: Romantic partners’ affirmations of nonbinary trans individuals. Int J Transgend [Internet]. 2019 [cited 2024 Jan 22];20(2–3):315. http://pmc/articles/PMC6831023/10.1080/15532739.2018.1496867 PMC6831023 32999616

[132] Grant R, Amos N, Lin A, Cook T, Hill AO, Pang K et al. Mental health and wellbeing outcomes associated with social, medical, and legal gender affirmation among trans young people in Australia. Int J Transgend Health. 2024. 10.1080/26895269.2024.2366881 PMC12857698 41625009

[133] Kalisch R, Russo SJ, Müller MB. Neurobiology and systems biology of stress resilience. https://doi.org/10.1152/physrev000422023 [Internet]. 2024 Mar 14 [cited 2024 Mar 16]; 10.1152/physrev.00042.2023 10.1152/physrev.00042.2023 PMC11381009 38483288

[134] Kalisch R, Müller MB, Tüscher O. A conceptual framework for the neurobiological study of resilience. Behav Brain Sci. 2015;38:e92. 25158686 10.1017/S0140525X1400082X

[135] Yehuda R, Flory JD, Southwick S, Charney DS. Developing an agenda for translational studies of resilience and vulnerability following trauma exposure. In: Annals of the New York Academy of Sciences; 2006. 10.1196/annals.1364.028 16891584

[136] Russo SJ. Neurobiology of Resilience. Nat Neurosci. 2012;15(11):1475–84. 23064380 10.1038/nn.3234 PMC3580862

[137] Schultebraucks K, Choi KW, Galatzer-Levy IR, Bonanno GA. Discriminating Heterogeneous Trajectories of Resilience and Depression After Major Life Stressors Using Polygenic Scores. JAMA Psychiatry [Internet]. 2021 Jul 1 [cited 2022 Sep 28];78(7):744–52. https://jamanetwork.com/journals/jamapsychiatry/fullarticle/2778021 10.1001/jamapsychiatry.2021.0228 PMC8014197 33787853

[138] Narayan AJ, Rivera LM, Bernstein RE, Harris WW, Lieberman AF. Positive childhood experiences predict less psychopathology and stress in pregnant women with childhood adversity: a pilot study of the benevolent childhood experiences (BCEs) scale. Child Abuse Negl. 2018;78:19–30. 28992958 10.1016/j.chiabu.2017.09.022

[139] Stadler G, Chesaniuk M, Haering S, Roseman J, Straßburger VM, Martina S, et al. Diversified innovations in the health sciences: proposal for a diversity minimal item set (DiMIS). Sustain Chem Pharm. 2023;33:101072.

[140] Potter AS, Dube SL, Barrios LC, Bookheimer S, Espinoza A, Feldstein Ewing SW et al. Measurement of gender and sexuality in the Adolescent Brain Cognitive Development (ABCD) study. Dev Cogn Neurosci [Internet]. 2022 Feb 1 [cited 2024 Jan 18];53. https://pubmed.ncbi.nlm.nih.gov/35026661/10.1016/j.dcn.2022.101057 PMC8759998 35026661

[141] Dhamala E, Yeo BTT, Holmes AJ. One size does not fit all: methodological considerations for brain-based predictive modeling in Psychiatry. Biol Psychiatry. 2023;93(8):717–28. 36577634 10.1016/j.biopsych.2022.09.024

[142] Shapiro JR, Klein SL, Morgan R. Stop ‘controlling’ for sex and gender in global health research. BMJ Glob Health [Internet]. 2021 Apr 1 [cited 2024 Jan 11];6(4):e005714. https://gh.bmj.com/content/6/4/e005714 10.1136/bmjgh-2021-005714 PMC8048018 33846145

[143] Wierenga LM, Ruigrok A, Aksnes ER, Barth C, Beck D, Burke S et al. Recommendations for a Better Understanding of Sex and Gender in the Neuroscience of Mental Health. Biological Psychiatry Global Open Science [Internet]. 2024 Mar 1 [cited 2024 Jul 7];4(2):100283. http://www.bpsgos.org/article/S2667174323001611/fulltext 10.1016/j.bpsgos.2023.100283 PMC10837069 38312851

[144] Masten AS. Ordinary magic: resilience processes in development. Am Psychol. 2001;56(3):227–38. 11315249 10.1037//0003-066x.56.3.227

[145] Kopal J, Uddin LQ, Bzdok D. The end game: respecting major sources of population diversity. Nature Methods. 2023 20:8 [Internet]. 2023 Mar 3 [cited 2023 Dec 4];20(8):1122–8. https://www.nature.com/articles/s41592-023-01812-3 10.1038/s41592-023-01812-3 36869122

[146] Dhamala E, Rong Ooi LQ, Chen J, Ricard JA, Berkeley E, Chopra S, et al. Brain-based predictions of Psychiatric illness–linked behaviors across the sexes. Biol Psychiatry. 2023;94(6):479–91. 37031778 10.1016/j.biopsych.2023.03.025 PMC10524434

[147] Dong D, Pizzagalli DA, Bolton TAW, Ironside M, Zhang X, Li C et al. Sex-specific resting state brain network dynamics in patients with major depressive disorder. Neuropsychopharmacology 2024 [Internet]. 2024 Jan 13 [cited 2024 Jan 15];1–8. https://www.nature.com/articles/s41386-024-01799-1 10.1038/s41386-024-01799-1 PMC10948777 38218921

[148] Rechlin RK, Splinter TFL, Hodges TE, Albert AY, Galea LAM. An analysis of neuroscience and psychiatry papers published from 2009 and 2019 outlines opportunities for increasing discovery of sex differences. Nature Communications 2022 13:1 [Internet]. 2022 Apr 19 [cited 2024 Jan 18];13(1):1–14. https://www.nature.com/articles/s41467-022-29903-3 10.1038/s41467-022-29903-3 PMC9018784 35440664

[149] Jacobs EG. Leveraging precision neuroimaging to advance women’s brain health. Nature Mental Health 2023 1:10 [Internet]. 2023 Oct 5 [cited 2024 Jan 11];1(10):700–1. https://www.nature.com/articles/s44220-023-00098-w

